# First Draft Genome Sequences of *Neisseria* sp. Strain 83E34 and *Neisseria* sp. Strain 74A18*,* Previously Identified as CDC Eugonic Fermenter 4b Species

**DOI:** 10.1128/genomeA.01277-16

**Published:** 2016-11-10

**Authors:** Alexander L. Greninger, Jessica Streithorst, Charles Y. Chiu, Steve Miller

**Affiliations:** Department of Laboratory Medicine, UCSF, San Francisco, California, USA

## Abstract

We report the first draft genome sequences of two isolates previously classified as CDC EF-4b species, *Neisseria* sp. 83E34 and *Neisseria* sp. 74A18. Both strains were isolated from patients with animal bites and likely constitute novel genomospecies with average nucleotide identities of <95% to other sequenced strains.

## GENOME ANNOUNCEMENT

CDC eugonic fermenter 4b (EF-4b) is a designation of Gram-negative bacteria that are members of animal oral flora and are typically isolated from animal bite wounds in humans (1). In 2006, this group was identified as *Neisseria zoodegmatis* based on 16S sequence and biochemical testing (1–3). Only five isolates from human infections have been reported since the taxonomical classification of *Neisseria zoodegmatis*, while >30 isolates have been collected from CDC EF-4b bacteria (1, 4, 5). CDC EF-4b has also been cultured from a case of infectious tenosynovitis due to a Siberian tiger bite (6). To date, the 16S sequence is the only available nucleotide sequence for *Neisseria zoodegmatis.*

We sequenced the first draft genomes of two bacterial isolates that had been identified as CDC group EF-4b from the University of California, San Francisco (UCSF) microbiology lab. Isolate 74A18 was isolated from a right-hand index finger culture of a patient with flexor tenosynovitis following a dog bite. Isolate 83E34 was isolated from a left-lateral-hand culture of a patient with cellulitis following a cate bite. Both patients also had *Pasteurella multocida* isolated concurrently from the wound cultures.

DNA was extracted using the Qiagen EZ1 DNA tissue kit. Nextera XT paired-end and Nextera mate-pair libraries were sequenced at 250/350 bp and 2 × 80 bp on an Illumina MiSeq, respectively, and on an Oxford Nanopore MinION sequencer. Illumina sequences were adapter and quality (Q20) trimmed using cutadapt or NxTrim, *de novo* assembled using SPAdes version 3.5, metagenomically screened for contaminating sequence with SURPI, and annotated via Prokka version 1.1 (7–12). A total of 6,878,276 (74A18) and 9,380,478 (83E34) paired-end reads and 4,052,930 (74A18) and 5,428,958 (83E34) mate-pair reads were recovered after trimming. *De novo* assembly yielded 40 contigs totaling 2,419,010 bp, with an *N*_50_ of 233,412 bp for *Neisseria* sp. 83E34, while *Neisseria* sp. 74A18 yielded 88 contigs totaling 2,572,932 bp, with an *N*_50_ of 53,927 bp.

BLASTN analysis of the 16S sequence from *Neisseria* sp. 74A18 showed 98.6% identity to *Neisseria shayeganii* clone TM092 (accession no. KM462144) and 98.1% identity to *Neisseria zoodegmatis* strain N15a (accession no. JQ979306). BLASTN analysis of the 16S sequence from *Neisseria* sp. 83E34 demonstrated 99.3% identity to *Neisseria canis* oral taxon 137 (accession no. JN713302), 98.2% identity to *Neisseria shayeganii* clone TM092 (accession no. KM462144), and 96.7% identity to *Neisseria zoodegmatis* strain N15a (accession no. JQ979306). However, alignment of *Neisseria* sp. 83E34 to *Neisseria canis* housekeeping genes revealed poor alignment (84.7% for *rpoB* [accession no. KM438030] and 88.4% for *cpn60* [accession no. KJ872773]), suggesting it may not be a strain of *Neisseria canis*. Pairwise whole-genome alignment by LASTZ revealed 89.3% identity between our two sequenced species and 93.9% identity between *Neisseria* sp. 83E34 and *Neisseria wadsworthii* 9715 (WGS AGAZ01), the closest whole genome available (13). These data are consistent with a high degree of genomic variability within related *Neisseria* spp. and isolates previously identified as CDC EF-4 members (14). Further sequencing of EF-4b species, including *Neisseria zoodegmatis*, will be needed to fully classify these species.

### Accession number(s).

These whole-genome shotgun projects have been deposited at DDBJ/ENA/GenBank under the accession numbers LGYH00000000 (*Neisseria* sp. 83E34) and LGZA00000000 (*Neisseria* sp. 74A18).
